# Accuracy of Artificial Intelligence Based Chatbots in Analyzing Orthopedic Pathologies: An Experimental Multi-Observer Analysis

**DOI:** 10.3390/diagnostics15020221

**Published:** 2025-01-19

**Authors:** Tobias Gehlen, Theresa Joost, Philipp Solbrig, Katharina Stahnke, Robert Zahn, Markus Jahn, Dominik Adl Amini, David Alexander Back

**Affiliations:** 1 Center for Musculoskeletal Surgery, Charité-Universitätsmedizin Berlin, Corporate Member of Freie Universität Berlin, Humboldt-Universität zu Berlin, and Berlin Institute of Health, 13353 Berlin, Germany; t.gehlen@ma-praxis.de (T.G.); katharina.stahnke@charite.de (K.S.); robert.zahn@charite.de (R.Z.); markus.jahn@charite.de (M.J.); dominik.adl-amini@charite.de (D.A.A.); 2 Move Ahead-Foot Ankle and Sportsclinic, 10117 Berlin, Germany; 3 Sports Medicine & Sports Orthopedics, University Outpatient Clinic, University of Potsdam, 14469 Potsdam, Germany

**Keywords:** orthopedics, traumatology, chatbots, artificial intelligence, symptoms, mobile health

## Abstract

**Background and Objective:** The rapid development of artificial intelligence (AI) is impacting the medical sector by offering new possibilities for faster and more accurate diagnoses. Symptom checker apps show potential for supporting patient decision-making in this regard. Whether the AI-based decision-making of symptom checker apps shows better performance in diagnostic accuracy and urgency assessment compared to physicians remains unclear. Therefore, this study aimed to investigate the performance of existing symptom checker apps in orthopedic and traumatology cases compared to physicians in the field. **Methods:** 30 fictitious case vignettes of common conditions in trauma surgery and orthopedics were retrospectively examined by four orthopedic and traumatology specialists and four different symptom checker apps for diagnostic accuracy and the recommended urgency of measures. Based on the estimation provided by the doctors and the individual symptom checker apps, the percentage of correct diagnoses and appropriate assessments of treatment urgency was calculated in mean and standard deviation [SD] in [%]. Data were analyzed statistically for accuracy and correlation between the apps and physicians using a nonparametric Spearman’s correlation test (*p* < 0.05). **Results:** The physicians provided the correct diagnosis in 84.4 ± 18.4% of cases (range: 53.3 to 96.7%), and the symptom checker apps in 35.8 ± 1.0% of cases (range: 26.7 to 54.2%). The agreement in the accuracy of the diagnoses varied from low to high (Physicians vs. Physicians: Spearman’s ρ: 0.143 to 0.538; Physicians vs. Apps: Spearman’s ρ: 0.007 to 0.358) depending on the different physicians and apps. In relation to the whole population, the physicians correctly assessed the urgency level in 70.0 ± 4.7% (range: 66.7 to 73.3%) and the apps in 20.6 ± 5.6% (range: 10.8 to 37.5%) of cases. The agreement on the accuracy of estimating urgency levels was moderate to high between and within physicians and individual apps. **Conclusions:** AI-based symptom checker apps for diagnosis in orthopedics and traumatology do not yet provide a more accurate analysis regarding diagnosis and urgency evaluation than physicians. However, there is a broad variation in the accuracy between different digital tools. Altogether, this field of AI application shows excellent potential and should be further examined in future studies.

## 1. Introduction

The rapid development of artificial intelligence (AI) has impacted the medical sector in recent years. It offers new possibilities, including faster and more accurate diagnostics with the potential to increase efficiency in the healthcare system [1,2]. Applications in radiology, for example, enable the automatic evaluation of X-ray images with previously trained systems [3,4]. Other research areas for the potential application of AI in medicine include oncology [5,6,7], cardiology [8,9,10], and gastroenterology [11,12]. Common approaches include using AI to improve various phases of patient care, from research and diagnosis to selecting suitable therapy [8]. Another, albeit more nuanced, field is the market for mobile health applications (apps) [9]. Frequently used apps for monitoring diseases [1] or fitness also provide personalized health information and recommendations [9]. Simultaneously, AI app solutions for patients have gained importance [13,14]. Many of these are primarily designed for non-professionals to interact with their users via individual question trees such as chatbots and so-called symptom checker apps [13,14,15,16]. After entering their symptoms, some apps provide patients with a diagnosis probability and advice on how they should proceed [13,14,15,16]. The prospective benefits of such technologies are manifold. Particularly in rural areas, where a shortage of doctors and specialists leads to long waiting times, prior triage using a reliable app could be of great benefit in directing patient flows [17]. The first support systems for medical staff in emergency rooms are already being tested [18,19]. In the years since the coronavirus spread, diagnostic apps have also made it possible to monitor the course of the pandemic [2]. However, the accuracy of AI-based statements [20,21] is still unknown for many medical fields. It poses a potential problem, as they cannot fully reflect a human assessment of a patient’s overall appearance based on experience and intuition [20,21,22,23,24,25]. In orthopedics and traumatology, comparative studies on symptom checkers and statements on their accuracy have been lacking. Therefore, this study aimed to investigate the performance of different market-available symptom checker apps using reconstructed ideal–typical disease cases by (a) evaluating their ability to recognize clinical pathologies in orthopedics and traumatology accurately and (b) classifying their respective clinical urgency.

## 2. Material and Methods

### 2.1. Study Design

Fictive case vignettes of common clinical pathologies in traumatology and orthopedics were created for this case vignette study. The cases were then assigned to four volunteer orthopedic surgeons, who were asked to indicate the three most likely diagnoses and an urgency recommendation for each case. The data were manually documented, transferred to an Excel file (Microsoft Corp., Redmond, WA, USA), and coded for analysis. In the following, the same orthopedic surgeons entered the case vignettes into four different symptom checker apps (see below). Data were saved digitally, manually transferred to an Excel file, and coded for further statistical evaluation. The study was conducted in compliance with the Declaration of Helsinki. Since no personal patient data were used, the need for an approval statement from the local ethical committee was obsolete.

### 2.2. Case Vignettes

Specialist physicians selected the pathologies of the fictive case vignettes (*n* = 30). They included common pathologies relating to the areas of the foot and ankle (*n* = 5), hand (*n* = 5), spine (*n* = 5), knee (*n* = 5), hip (*n* = 5), and shoulder (*n* = 5). Based on current literature, the cases were created under specialist supervision with current anamnesis, relevant previous history, accident history (if applicable), social anamnesis, medication anamnesis, and vegetative anamnesis with possible previous illnesses.

### 2.3. Selection of Symptom Checkers

The selection of symptom checker apps was based on a literature review. The chosen tools were Ada Health (version 3.13.9, Ada Health GmbH, Berlin, Germany), Babylon (version 4.35.0, Babylon Health, London, UK), Symptoma (version 2.1, Symptoma GmbH, Attersee, Austria), and Symptomate (version 2.4, Infermedica Sp. Z o.o, Wroclaw, Poland), accessible on mobile devices (iOS, iPhone 7 and iPad mini, Apple Inc., Cupertino, CA, USA) with specially created user accounts. Ada, Symptoma, and Symptomate were used to detect diagnosis and estimate urgency. The Babylon app was additionally and solely used to assess urgency, as this app is widely used in clinical practice.

### 2.4. Data Rating and Grading for Evaluation

**Figure 1:**
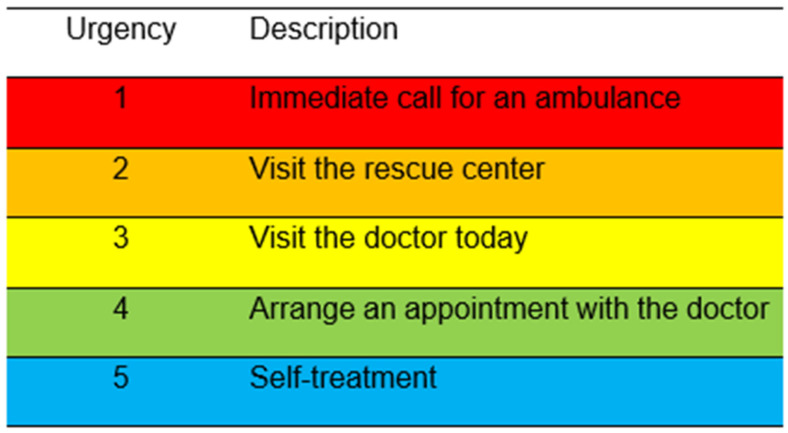
Urgency recommendations staged from 1 to 5.

The diagnosis and urgency recommendation of the case vignettes set during creation by an independent specialist was defined as the gold standard. For the diagnoses, single points were awarded for the answers (correct diagnosis = 1 point; incorrect diagnosis = 0 points). Three suggestions per case and doctor were allowed. In the evaluation made by the apps, only the first three diagnoses were included if more were provided (D1, D2, D3). Points were also awarded based on the number of attempts required to reach the correct diagnosis (1 attempt = 3 points, 2 attempts = 2 points, 3 attempts = 1 point). No points were scored if the correct diagnosis was not made even after 3 attempts. The urgency recommendations by the experts and as far as given by the single apps were staged according to the classification table depicted in Figure 1.

### 2.5. Statistical Analysis

The accuracy of the correct diagnosis and the number of attempts required were analyzed descriptively (mean and standard deviation [SD] in [%]) for both the doctors and the apps. In addition, the correlation of the responses (a) among the doctors, (b) between doctor vs. apps (each for doctor 1 to 4 and respective apps 1 to 4), and (c) between the results within the respective apps was determined using the nonparametric Spearman’s correlation test (*p* < 0.005). The assessment of the degree of correlation was categorized according to Cohen et al. Thus, |ρ| = 0.10 indicates a small correlation, |ρ| = 0.30 a moderate correlation, and |ρ| = 0.50 a strong correlation [26].

## 3. Results

Based on the given 30 vignettes, altogether, *n* = 600 single cases with *n* = 120 doctor case ratings and *n* = 480 app case ratings were created and analyzed.

### 3.1. Accuracy of Diagnoses

**Figure 2:**
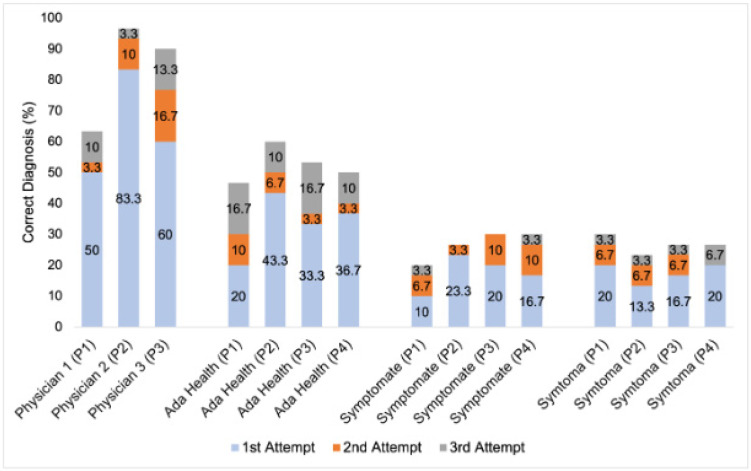
Percentage of correct diagnoses via physicians (*n* = 3 diagnosis) and symptom checker apps (*n* = 4) based on case vignettes (*n* = 30).

On average, the doctors provided the correct diagnosis in 84.4 ± 18.4% of cases (range: 53.3 to 96.7%) and the symptom checker apps in 35.8 ± 1.0% of cases. The symptom checker app Ada provided the correct diagnosis in 54.2 ± 4.2% of cases (range: 50.0 to 60.0%), Symptomate in 26.7 ± 4.7% (20.0 to 30.0%), and Symptoma in 26.7 ± 2.7% (23.3 to 30.0%) of the evaluated cases. A detailed overview of the results can be found in Figure 2.

Table 1 shows the degree of agreement between the physicians regarding the accuracy of the correct diagnosis. Spearman’s correlation test was used to calculate this agreement.

The correlation of the diagnostic results between Doctor 1 and the symptom checker apps varied from low to moderate. The correlation of the respective results between Doctors 2, 3, and 4 and the respective symptom checker apps was low. Detailed results are shown in Table 2.

### 3.2. Urgency

Two of the four physicians assessed the urgency of the medical treatment of the cases based on the correct estimated diagnosis. On average, they correctly assessed the urgency level at 75.9 ± 0.8% (range: 75.9 to 76.9%). In relation to the total population (*n* = 30), the physicians’ success rate is 70 ± 4.7% (range: 66.7 to 73.3%). In 10.9 ± 0.8% (range: 10.3 to 11.5%) of the evaluated cases, the degree of urgency was assessed as too low, and in 12.7 ± 1.6% (range: 11.5 to 13.8%), it was too high.

The data from 3 (Ada Health, Babylon, and Symptomate) symptom checker apps were also evaluated regarding the correctness of the urgency assessment where correct diagnosis was provided beforehand. On average, the level of urgency was correctly assessed in 37.6 ± 10.3% (range: 21.0 to 50.7%) of cases. In relation to the total population (*n* = 30), the apps’ success rate is 20.6 ± 5.6% (range: 10.8 to 37.5%). On average, the success rate was 21.9 ± 2.0% for Ada Health, 41.0 ± 17% for Babylon, and 51.0 ± 21.8% for Symptomate. In relation to the total population (*n* = 30), Ada’s success rate was 10.8 ± 1.7% (range: 10.0 to 13.3%), Babylon’s success rate was 37.5 ± 12.9 (range: 20.0 to 50.0%), and Symptomate’s success rate was 13.3 ± 6.1% (range: 6.7 to 20.0%). Ada Health rated the urgency as too low in 67.0 ± 7.9%, Babylon in 44.4 ± 19.2%, and Symptomate in 25.0 ± 12.8% of cases. Meanwhile, Ada rated the urgency too high in 18.0 ± 4.5%, Babylon in 14.4 ± 2.7%, and Symptomate in 29.9 ± 11.9% of cases. Details are provided in Table 3.

There is no agreement in the accuracy of the urgency evaluation between the physicians calculated using Spearman’s correlation test (Spearman’s ρ = 0.000, *p* not available).

The correlation of the accuracy of the urgency evaluation of the case vignettes entered by the four doctors varied from zero to perfect within the individual apps.

The correlation of the accuracy of the urgency evaluation of the case vignettes entered by the four physicians varied from moderate to strong between the apps. Detailed results are shown in Table 4.

## 4. Discussion

The study aimed to investigate the performance of different market-available symptom checker apps using reconstructed ideal–typical disease cases by (a) evaluating their ability to recognize clinical pathologies in orthopedics and traumatology accurately and (b) classifying their respective clinical urgency compared to specialized physicians.

Even though the use of algorithms with artificial intelligence (AI) is making increasing progress in medicine and promises to support and improve healthcare in the future significantly, the results of this study indicate no superiority regarding the accuracy of diagnosis and estimation of urgency in decision-making compared to physicians [6,8,9].

The results reveal that physicians were, on average, around 75% accurate in making a correct diagnosis. However, there were fluctuations of 53–96% between doctors.

On average, the success rate for symptom checker apps was significantly lower. Here, considerable differences were found between the individual providers. Ada Health achieved 54% and thus delivered comparable results to the doctor with the lowest performance. Symptoma and Symptomate performed poorly, with an accuracy of only 27%.

The above-mentioned apps delivered significantly better results in the study by Gilbert et al., in which an accuracy of 99% was achieved for Ada Health, for example [27]. One of the main differences here lies in the medical field. In that study, the app was used by general practitioners and not doctors specializing in orthopedics and traumatology. In our study, only diagnoses relating to the musculoskeletal system were evaluated. Therefore, it can be assumed that AI is not yet sufficiently developed in this special medical field to achieve values nearly as good as those of a specialist. The correlation between the accuracy of the diagnosis varied within the medical profession. The agreement of accuracy of diagnosis between physicians 1 and 2 and physicians 1 and 3 showed a significantly high correlation, whereas, between physician 2 and physician 3, it was low. Relevant differences could be found between the doctors and apps and the apps themselves. In 2016, Bisson et al. showed results similar to our study [15]. Here, 328 patients with knee pain were diagnosed using a web-based symptom checker application, and the results were compared with those of rating doctors. The accuracy reached 58%, which seems comparable to the results of the best app in the study presented here.

Significant differences could be detected between the apps, particularly in diagnostic accuracy, with Ada Health achieving the best results, higher than Symptomate or Symptoma. Ceney et al. also examined symptom checkers tailored to emergencies in Australia [28]. Here, Ada Health also proved to be the most reliable app. The range for the accuracy of diagnoses in this study was 22% and 84%. It was concluded that the accuracy of diagnosis must be higher in the emergency sector, in particular, than in other medical fields, where reliability does not necessarily determine the patient’s outcome.

Regarding urgency recommendations, physicians performed significantly better than the apps. The apps tended to give rather conservative recommendations and classified the respective results, on average, one urgency level lower. The urgency assessment by both physicians was consistently high, at around 75%. They were too high or too low in equal proportions of 10–13%. There were again clear differences in the apps in this study. Symptomate was the leader here, with 51% accuracy; Babylon was at 41%; and the weakest and least accurate app was Ada Health, with an average of 21% (outlier when entering doctor 2; there, accuracy was 60%).

The accuracy of the urgency assessment in this study is based only on the correct diagnoses. As mentioned above, Symptomate performed significantly worse here than Ada Health. However, despite the small number of cases, Symptomate had a high level of accuracy concerning the urgency recommendation compared to Babylon and Ada Health if the diagnosis was correct. Chambers et al. found that online symptom checkers made correct triage recommendations in 58% of cases with different platforms. The study showed that around 20–30% of recommendations resulted in over-triage, while under-triage occurred in 10–15% of cases [29].

One of the limitations of this work is that it only represents a snapshot of the respective versions of the selected apps, as updates and enhancements to the AI can lead to improvements at short intervals. Furthermore, data evaluation of the urgency recommendations of the apps was complex, as each provider used its own system to classify urgency, which is why individual adjustments were necessary for this work. This also applied in the event that the apps asked questions that were not covered by the vignettes. In addition, only a few doctors participated in this study, so the small data set may have led to distortions in the results. More significant case numbers should be planned for future studies. In the future, case vignettes should be used repeatedly, along with newer versions of suitable symptom checkers. As AI algorithms’ capabilities increase, a rapid improvement in diagnostic functions is expected, maybe up to the equivalent of a doctor’s level [25,30,31,32]. These applications could then lead to a relief of existing structures by improving individual patient flow coordination, e.g., as part of patient-induced primary triage in many healthcare systems. Strict regulations and guidelines for protecting health data will be essential here.

## 5. Conclusions

In summary, the algorithm versions of digital AI symptom checkers used for diagnosis in orthopedics do not yet offer a more accurate analysis than specialist physicians for the fictitious case vignettes used. However, it could also be shown that there were already clear differences in accuracy between the products tested. When classifying the urgency of treatment, the apps tended to be more cautious than the human specialists. Further development and research will be necessary to exploit the potential of AI in diagnostics fully in the future.

## Figures and Tables

**Table 1:** Correlation in the evaluation of correct diagnosis between doctors.

	Spearman’s ρ	Significance (2-Tailed) ρ
Doctor 1 vs. Doctor 2	0.538	0.002
Doctor 1 vs. Doctor 3	0.388	0.034
Doctor 2 vs. Doctor 3	0.143	0.450

**Table 2:** Correlation between doctors and symptom checker apps.

	Spearman’s ρ	Significance (2-Tailed) ρ
Doctor 1 vs. Ada	0.036	0.849
Doctor 1 vs. Symptomate	0.358	0.052
Doctor 1 vs. Symptoma	0.037	0.846
Doctor 2 vs. Ada	0.007	0.969
Doctor 2 vs. Symptomate	n/a	n/a
Doctor 2 vs. Symptoma	0.267	0.154
Doctor 3 vs. Ada	−0.160	0.399
Doctor 3 vs. Symptomate	0.040	0.832
Doctor 3 vs. Symptoma	−0.086	0.653

**Table 3:** Percentage of correct diagnosis via doctors (*n* = 2) and symptom checker apps (*n* = 3).

	Correct Evaluation [%]	Incorrect Evaluation (Too Low) [%]	Incorrect Evaluation (Too High) [%]	Number of Cases [*n*]
Doctor 2	75.9	10.3	11.5	29
Doctor 3	76.9	13.8	11.5	26
Ada (Dr_1)	20.0	60.0	20.0	3
Ada (Dr_2)	60.0	43.3	6.7	18
Ada (Dr_3)	23.1	76.9	23.1	16
Ada (Dr_4)	18.8	68.8	12.5	16
Babylon (Dr_1)	22.2	66.7	11.1	27
Babylon (Dr_2)	36.7	50	13.3	30
Babylon (Dr_3)	43.3	40.0	16.7	30
Babylon (Dr_4)	62.5	20.8	16.7	24
Symptomate (Dr_1)	50.0	33.3	16.7	6
Symptomate (Dr_2)	75.0	0.0	25.0	6
Symptomate (Dr_3)	55.6	11.1	33.3	9
Symptomate (Dr_4)	22.2	33.3	44.4	9

**Table 4:** Correlation of accuracy in urgency evaluation between physicians (*n* = 4) and Symptom Checker apps (*n* = 3).

	Ada Health (P1)	Ada Health (P2)	Ada Health (P3)	Ada Health (P4)	Babylon (P1)	Babylon (P2)	Babylon (P3)	Babylon (P4)	Symptomate (P1)	Symptomate (P2)	Symptomate (P3)	Symptomate (P4)
Ada Health (P1)		0.671 * (12)	1.000 * (13)	0.604 * (14)	0.408 (14)				0.833 (4)			
Ada Health (P2)	0.671 * (12)		1.000 * (12)	1.000 * (14)		0.315 (18)				0.745 (5)		
Ada Health (P3)	1.000 * (13)	1.000 * (12)		1.000 * (12)			0.415 (16)				0.894 * (6)	
Ada Health (P4)	0.604 * (14)	1.000 * (14)	1.000 * (12)					0.669 * (14)				0.323 (5)
Babylon (P1)	0.408 (14)					0.563 * (27)	0.550 * (27)	0.660 * (21)	0.833 (6)			
Babylon (P2)		0.315 (18)			0.563 * (27)		0.474 * (30)	0.607 * (24)		0.690 (8)		
Babylon (P3)			0.415 (16)		0.550 * (27)	0.474 * (30)		0.724 * (24)			0.926 * (9)	
Babylon (P4)				0.669 * (14)	0.660 * (21)	0.607 * (24)	0.724 * (24)					0.567 (6)
Symptomate (P1)	0.833 (4)				0.833 (6)					0.000 * (4)	1.000 * (3)	1.000 * (3)
Symptomate (P2)		0.745 (5)				0.690 (8)			0.000 * (4)		0.791 (5)	0.577 (4)
Symptomate (P3)			0.894 * (6)				0.926 * (9)		1.000 * (3)	0.791 (5)		0.943 (4)
Symptomate (P4)				0.323 (5)				0.567 (6)	1.000 * (3)	0.577 (4)	0.943 (4)	

* = *p* < 0.05; P1: Physician 1, P2: Physician 2, P3: Physician 3, P4: Physician 4; green: strong correlation, blue: moderate correlation, orange: no correlation; number of cases in brackets.

## Data Availability

The Data can be provided by the corresponding author upon reasonable request.
